# Protocol for integrative subtyping of lower-grade gliomas using the SUMO pipeline

**DOI:** 10.1016/j.xpro.2021.101110

**Published:** 2022-01-19

**Authors:** Karolina Sienkiewicz, Aakrosh Ratan

**Affiliations:** 1 Center for Public Health Genomics, University of Virginia, Charlottesville, VA 22908, USA; 2 Department of Public Health Sciences, University of Virginia, Charlottesville, VA 22908, USA; 3 University of Virginia Cancer Center, Charlottesville, VA 22908, USA

**Keywords:** Bioinformatics, Cancer, Health Sciences, Genomics, Gene Expression

## Abstract

Grouping patients into subtypes with homogeneous molecular features can guide diagnosis and therapeutic interventions. SUMO is a computational pipeline that uses nonnegative matrix factorization of patient-similarity networks to integrate continuous multi-omic datasets for molecular subtyping of a disease. Here, we present a detailed protocol to demonstrate its use in determining subtypes of lower-grade gliomas by integrating gene expression, DNA methylation, and miRNA expression data from the TCGA-LGG cohort.

For complete details on the use and execution of this profile, please refer to Sienkiewicz et al. (2022).

## Highlights

• Protocol to run and interpret the results of SUMO pipeline on a glioma dataset
• SUMO performs factorization of patient similarity networks to determine subtypes
• Special focus on data preparation of RNA-seq, methylation, and miRNA data

## Graphical abstract

**Figure:**
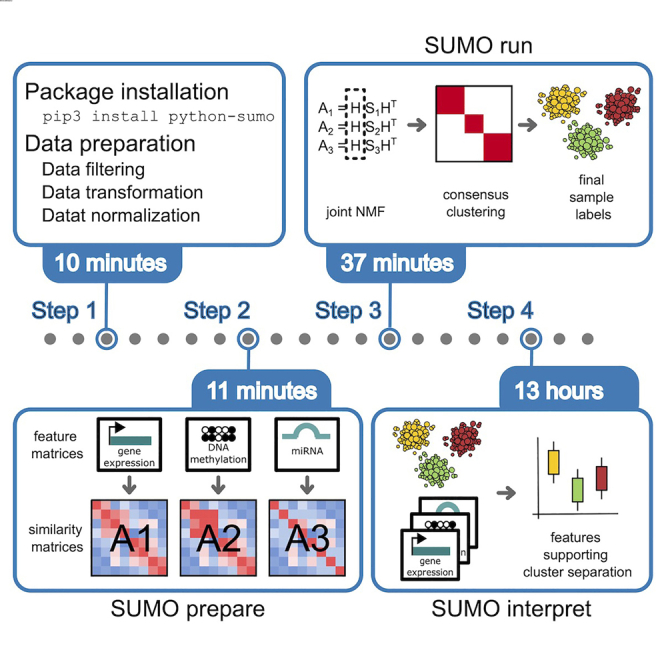

## Before you begin

SUMO can integrate continuous datasets that are represented using a feature matrix with samples as columns and features (e.g., gene, methylation probe) as rows. It can handle missing data in the feature matrices without imputation, which can be computationally intensive. The data pre-processing involves three steps that have to be performed before SUMO can create a patient-similarity network for each data type: (1) an optional data filtering, (2) a data-type-dependent transformation, and (3) normalization.

### Data filtering

1. We recommend removing non-informative features such as genes with zero counts in most samples, or features that do not vary over the samples. Such features do not affect the similarity between the patients and removing them can reduce the computational time required to construct the patient-similarity network.
2. Although SUMO can handle missing values in the feature matrices, we recommend removing features and samples with a large fraction of missing values (over 10%) unless it removes a significant portion of samples.

### Data transformation

3. Data transformation is used to handle data-type-specific bias and noise, and hence the steps are different for each data type. For count datasets such as RNA-seq, we recommend normalizing for library size, followed by a variance stabilizing transform (Anders and Huber, 2010) that removes the dependence of the variance on the mean of the counts. For methylation data, we recommend using M-values over beta-values, as M-values have reduced heteroscedasticity in the low and high methylation range (Du et al., 2010).

### Data normalization

4. In this normalization step, we perform feature standardization to make the value of each feature in the data be zero-mean and unit variance. This ensures that features contribute equally to the sample distance calculation.

## Key resources table

**Table:** 

REAGENT or RESOURCE	SOURCE	IDENTIFIER
**Deposited data**		

TCGA-LGG DNA methylation (level 3 Methylation 450k data)	UCSC Xena Browser	https://tcga-xena-hub.s3.us-east-1.amazonaws.com/download/TCGA.LGG.sampleMap%2FHumanMethylation450.gz
TCGA-LGG gene expression RNAseq (level 3 IlluminaHiSeq data)	UCSC Xena Browser	https://tcga-xena-hub.s3.us-east-1.amazonaws.com/download/TCGA.LGG.sampleMap%2FHiSeqV2.gz
TCGA-LGG miRNA mature strand expression RNAseq (level 3 IlluminaHiSeq data)	UCSC Xena Browser	https://tcga-xena-hub.s3.us-east-1.amazonaws.com/download/TCGA.LGG.sampleMap%2FmiRNA_HiSeq_gene.gz
TCGA-LGG curated survival data	UCSC Xena Browser	https://tcga-xena-hub.s3.us-east-1.amazonaws.com/download/survival%2FLGG_survival.txt

**Software and algorithms**		

SUMO	Sienkiewicz et al., 2022	https://github.com/ratan-lab/sumo; https://pypi.org/project/python-sumo

**Other**		

SUMO package documentation	GitHub	https://python-sumo.readthedocs.io

## Materials and equipment

• Tabular files, where each file contains processed output from a single genomic assay. Rows and columns in each file are expected to represent the samples and the features (e.g., genes, methylation probes) from the assay. Internally, we represent these files as data matrices with samples as columns and features as rows and refer to them as feature matrices. In this protocol, we use gene expression, DNA methylation, and miRNA expression data for patients from the TCGA Lower-Grade Glioma (LGG) cohort (Cancer Genome Atlas Research Network et al., 2015). The accession links are available in the key resources table. Some of the pre-processing steps are different when normalized or aggregated public datasets are used. See before you begin for additional instructions and SUMO documentation (see key resources table) for alternative pre-processing code examples.
• Python v3.6+, pip (the package installer for Python), and other required packages. Required packages will be installed in the first step of the protocol. The authors used Python v3.6 and following versions of packages when writing this protocol:
  SUMO (python-sumo package) v0.2.7
  numpy v1.19.5
  pandas v1.1.5
  scikit-learn v0.24.2
  scipy v1.5.4
  lightgbm v3.2.1
  hyperopt v0.2.5
  shap v0.39
  numba v0.53.1
  seaborn v0.11.1
  matplotlib v3.3.4
• R, and R packages for pre-processing and downstream analysis after determination of molecular subtypes. Installation instructions for the required packages are included in the protocol. The authors used R v3.6.3 and the following versions of packages:
  tidyverse v1.2.1
  devtools v2.4.1
  annotables v0.1.91
  survival v3.2-7
  survminer v0.4.6
  reticulate v1.13
• Hardware Recommendations:
  operating system: GNU/Linux, Mac OS
  memory: 16GB required (due to a large methylation dataset used in the protocol),
  processors: 1 required, 8 recommended

For timing purposes, the authors used a single core on a local (16GB) GNU/Linux system for all protocol steps, except for computationally expensive Step 5, which was run on a computational cluster using 256GB of memory and 10 cores.

## Step-by-step method details

A typical workflow to subtype a cohort using SUMO requires four steps, and each of the steps corresponds to a `mode` in SUMO. The first step in the analysis is the construction of patient-similarity networks from the pre-processed feature matrices (sumo prepare). Next, the multiplex similarity network is jointly factorized to identify the molecular subtypes (sumo run). We can then assess the importance of each feature (e.g., genes and methylation probes) in driving each of the identified subtypes (sumo interpret). If another set of labels for the same patients is known, for example, based on the International Statistical Classification of Diseases and Related Health Problems (ICD), then we can compare the labels assigned by SUMO to those labels using several external evaluation metrics including purity, normalized mutual information (NMI), and adjusted Rand index (ARI) (sumo evaluate).

### Step 1: Package installation

**Timing: 1 min**

The SUMO command-line tool can be installed from the Python Packaging Index (PyPI), by executing the command below. Note that SUMO requires a Python 3.6+ version and corresponding pip (package installer), distributed together with Python. Here, we refer to pip as pip3 to avoid confusion with the default Python 2 specific version of pip.

1. Upgrade pip3 (to a >19.0 version) and the setuptools package:

pip3 install --upgrade pip setuptools

2. Install SUMO, by running the following command-line command:

pip3 install python-sumo

### Step 2: Data preparation

**Timing: 9 min**

See before you begin for an explanation of data pre-processing steps. In this protocol, the gene expression and miRNA expression feature matrices downloaded from XENA have already been log-normalized to remove the dependence of the variance on the mean of the counts. So, we omit the data transformation step in the vignette below. Here, we use R to pre-process the dataset (see SUMO documentation for examples of pre-processing scripts in python). In this protocol, we assume that the user has set their working directory to the directory with the files downloaded from Xena (See the key resources table). The working directory can be set by using the ‘setwd’ command in R.

3. Install and load the required R packages:

>install.packages(c("devtools", "tidyverse",

     "reticulate", "survival", "survminer"))

>devtools::install_github("stephenturner/annotables@v0.1.90")

>library(annotables)

>library(tidyverse)

4. Pre-processing of miRNA expression data:
  a. Load the data:
    >data <-
     read.table("TCGA.LGG.sampleMap%2FmiRNA_HiSeq_gene.gz",
     row.names = 1, header = TRUE, check.names = FALSE)
  b. SUMO calculates the distance between each pair of samples using the normalized counts assigned to the miRNAs. Since we are typically dealing with high-dimensional datasets, we filter out features with several missing values. Here, we remove features with more than 10 % missing values:
    >data <- data[rowSums(is.na(data)) <=
     round(dim(data)[2]∗0.1),]
  c. Scale the features to have zero-mean and unit variance. Also, remove the features with zero standard deviation:
    >normalize.matrix <- function(data.matrix) {
     num = data.matrix - rowMeans(data.matrix,
      na.rm = TRUE)
     should.keep = (apply(num, 1, function(x) sd(x,
        na.rm=TRUE)) != 0)
     return ((num / apply(num, 1, function(x) sd(x,
     na.rm=TRUE)))[should.keep,])
     }
    >data.norm <- normalize.matrix(data)
  d. Write the data matrix into a tab-delimited file:
    >write.table(data.norm, file="TCGA.LGG.miRNAexp.tsv",
     sep="\t", col.names = TRUE, row.names = TRUE)

5. Pre-processing of gene expression data:
  a. Load the data:
    >data <- read.table("TCGA.LGG.sampleMap%2FHiSeqV2.gz",
     row.names = 1, header = TRUE, check.names = FALSE)
  b. Filter out non-informative features and features with several missing values. Here, we remove features with more than 10 % missing values and features where less than two samples have FPKM over zero:
    >data <- data[rowSums(is.na(data)) <=
        round(dim(data)[2]∗0.1),]
    >data <- data[rowSums(data > 1) > 1,]
  c. (optional) Limit the feature matrix to protein-coding genes:
    >protein_coding <- tibble(symbol=rownames(data)) %>%
      left_join(grch38, by = "symbol") %>%
      select(symbol, biotype) %>%
      filter(biotype == "protein_coding") %>% pull(symbol) %>% unique()
    >data <- data[rownames(data) %in% protein_coding,]
  d. Scale the features to have zero-mean and unit variance:
    >data.norm <- normalize.matrix(data)
  e. Write the data matrix into a tab-delimited file:
    >write.table(data.norm, file="TCGA.LGG.mRNAexp.tsv",
     sep="\t", col.names = TRUE, row.names = TRUE)

6. Pre-processing of methylation data:
  a. Load the data:
    >data <-
    read.table("TCGA.LGG.sampleMap%2FHumanMethylation450.gz",
     row.names = 1, header = TRUE, check.names = FALSE)
  b. Filter out features with several missing values. Here, we remove features with more than 10 % missing values:
    >data <- data[rowSums(is.na(data)) <=
     round(dim(data)[2]∗0.1),]
  c. Convert the beta values to corresponding M values, as M values have reduced heteroscedasticity in the low and high methylation range (Du et al., 2010):
    >dataM <- log2(data + .Machine$double.eps)/(1 - data +
     .Machine$double.eps)
  d. Scale the features to have zero-mean and unit variance. Also, remove the features with zero standard deviation:
    >dataM.norm <- normalize.matrix(dataM)
  e. Write the data matrix into a tab-delimited file:
    >write.table(dataM.norm, file="TCGA.LGG.met.tsv",
     sep="\t", col.names = TRUE, row.names = TRUE)

### Step 3: Creating similarity networks

**Timing: 11 min**

In this step, each of the pre-processed feature matrices is used to calculate data-type-specific distances between the samples. Next, the distances are converted to similarities between samples. Each similarity matrix corresponding to a different data type is then used as an adjacency matrix to construct a “layer” of a multiplex similarity network (see (Sienkiewicz et al., 2020) for more details about the method).

***Note:*** We assume that SUMO is run in the directory with the pre-processed files.

7. Use the following command to run SUMO on the pre-processed files:
  sumo prepare
  TCGA.LGG.mRNAexp.tsv,TCGA.LGG.met.tsv,TCGA.LGG.miRNAexp.tsv
  prepared.LGG.npz
  a. The required positional arguments of SUMO are:
    i “prepare” - selected sumo `mode` which uses the pre-processed feature matrices to construct the similarity networks,
    ii “infile1,infile2,…” - comma-delimited list of files containing standardized feature matrices, with samples in columns and features in rows (here: TCGA.LGG.mRNAexp.tsv,TCGA.LGG.met.tsv,TCGA.LGG.miRNAexp.tsv),
    iii “outfile.npz” - path to the output file in .npz format, which is a zipped archive of files named after the variable they contain (here: prepared.LGG.npz).
  b. Some of the optional arguments for SUMO in `prepare` mode include:
    i “-atol” - SUMO checks if the data was standardized by assessing the mean and standard deviation of each feature. This parameter allows the user to specify an allowed tolerance level in this test. In this protocol, we increase it to include the sparse miRNA expression matrix, with a smaller number of features (see Troubleshooting problem 1 for more details).
    ii “-method” – distance to be used in calculation of sample-sample similarity (here we use the default Euclidean distance, which is appropriate for log-normalized counts and M-values used in methylation data),
    iii “-k” and “-alpha” – hyperparameters that affect the distribution of values in the similarity matrix (see (Sienkiewicz et al., 2020) for more details),
    iv “-log” and “-logfile” - arguments governing the logging level and the optional path to save the logging file,
    v “-plot” - path to save the heatmaps that show the similarity matrix for each data type (the default behavior is to display these plots in an interactive mode on the screen).
  c. The above command creates a multiplex network file in form of a zipped NumPy archive containing:
    i the pairwise sample similarity matrices organized as network adjacency matrices in order of input files (arrays indexed: “0”, “1”, “2”),
    ii input feature matrices, after additional internal sumo filtering (arrays indexed: “f0”, “f1”, “f2”),
    iii an organized list of sample identifiers in the order corresponding to rows/columns of adjacency matrices (the “sample” array).

### Step 4: Detecting molecular subtypes

**Timing: 37 min**

The goal of this step is to determine the molecular subtypes in the cohort using the sample similarities encoded in the three data types. SUMO performs a joint non-negative matrix tri-factorization of the similarity networks to determine a representation of the samples in a lower rank subspace, which is then used to assign class labels. The factorization is performed several times on various subsets of samples, and SUMO uses consensus clustering to determine the final labels. Non-negative factorization assumes that a single basis vector can represent each cluster and different clusters correspond to different basis vectors. This makes estimating the optimal factorization rank (equivalent to the number of clusters in the dataset) a challenging problem. We recommend running SUMO on a range of possible values and utilizing clustering metrics calculated by SUMO to select the optimal number, as detailed below.

**Figure 1:**
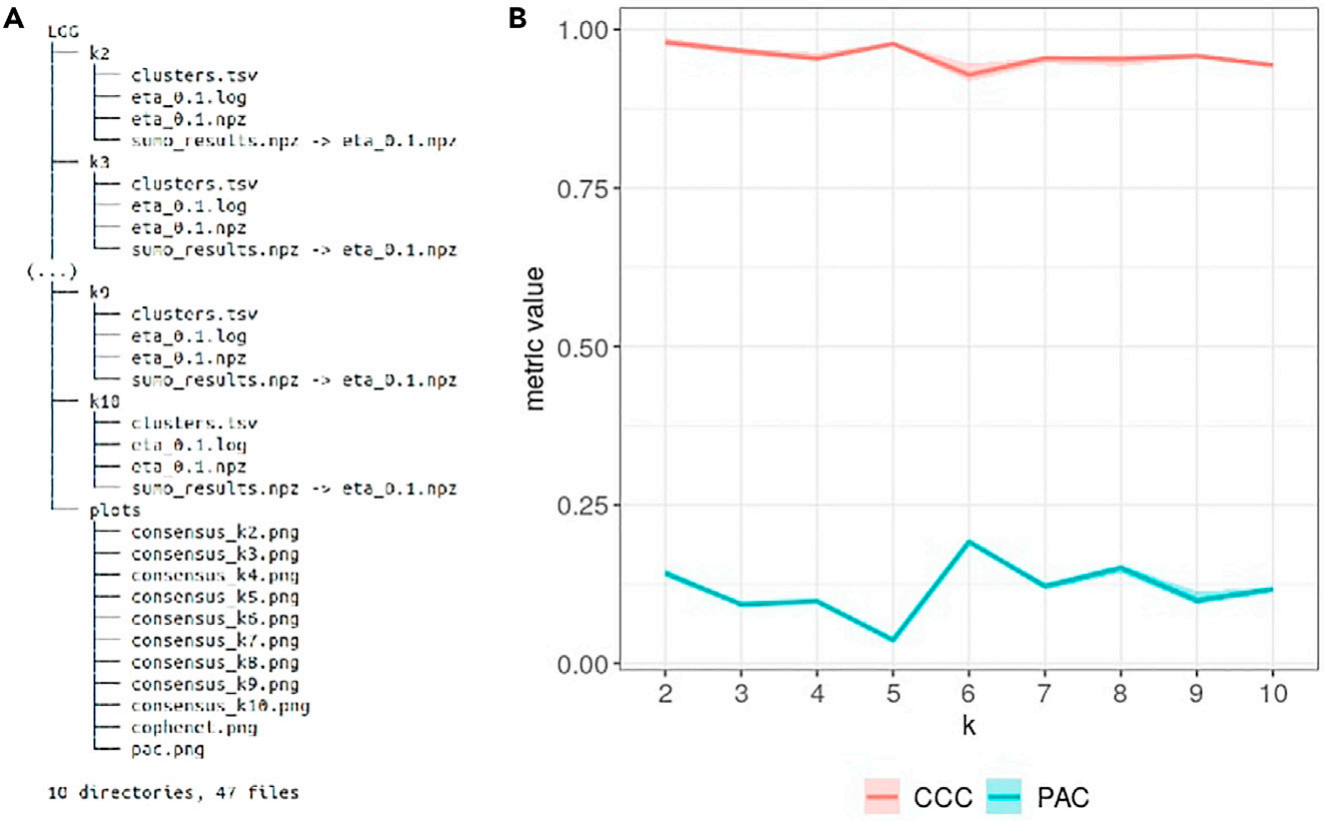
Output of `sumo run` (A) The structure of the output directory generated in Step 4 of the protocol. (B) Two internal clustering stability metrics calculated by SUMO: proportion of ambiguously clustered pairs (PAC) and cophenetic correlation coefficient (CCC). The optimal cluster rank is characterized by a low PAC value and a high CCC value.

8. Use the following command to run SUMO on the output from the last step:
  sumo run prepared.LGG.npz 2,10 LGG
  a. The required positional arguments of SUMO are:
    i “run” - selected sumo `mode` to subtype samples,
    ii “infile.npz” - output .npz file from the last step,
    iii “k” - either one value when the number of clusters is known or a comma-delimited range of values to run SUMO on a range of possible values for the number of clusters (here: 2,10 which will run SUMO for 2, 3, 4, …, 10 clusters),
    iv “outdir” - path to the output directory (here: “LGG”).
  b. Some of the optional arguments in this mode include:
    i “-log” and “-logfile” - arguments governing the logging level and optional path to save the logging file (using “-log DEBUG” flag results in saving additional arrays in the output files),
    ii “-max_iter“ and “-tol” - parameters governing the stopping criterion for the factorization: the maximum number of steps that should be taken by the solver in an attempt to minimize the cost function, and the minimum difference in the costs determined in consecutive steps before stopping,
    iii “-sparsity” - either one value or comma-delimited list of sparsity penalty (“ɳ”/”eta”) values (if multiple values are specified, SUMO runs factorization with different values and automatically select the best, by assessing within-cluster similarities; see (Sienkiewicz et al., 2020) for more details),
    iv “-t” number of computational threads.
  c. The output directory includes a sub-directory with the cluster assignments and information for each “k” (see Figure 1A). Every sub-directory contains:
    i a .log file and a .npz file with factorization results for each “eta” value,
    ii “sumo_results.npz” - a hyperlink to factorization result corresponding to the best “eta”,
    iii “clusters.tsv” - a file with the assigned sample labels.
      Additionally, SUMO creates a sub-directory “plots” with several plots that can assist in the selection of the optimal number of subtypes in the dataset.
  d. Each .npz file with the factorization results for given (“k”, “eta”) pair contains:
    i two internal metrics, namely the proportion of ambiguously clustered pairs (PAC) (Şenbabaoğlu et al., 2014) and cophenetic correlation coefficient (Hutchins et al., 2008). Each metric is calculated multiple times on subsets of samples and the results are saved in the “pac” and “cophenet” arrays,
    ii quality metric assessing the within-cluster similarities of final sample groups (“quality” array), used for sparsity parameter selection,
    iii consensus matrix resulting from running the factorization multiple times on subsets of samples (“unfiltered_consensus” array) and its filtered copy used for assignment of final sample labels (“consensus” array)

9. Select the optimal number of clusters/factorization rank.
  a. (recommended) Inspect the “cophenet.png” and “pac.png” files in created “LGG/plots” sub-directory. The created plots show the distribution of two popular clustering metrics: the cophenetic correlation coefficient (CCC) and the proportion of ambiguously clustered pairs (PAC). We recommend selecting the factorization rank for which the CCC is high (over 0.95) and PAC score is less than 0.1.
  b. (extended) Use the following commands to plot both ranks together in R (see Figure 1B):
    i. load required R packages:
      >library(tidyverse)
      >library(reticulate)
    ii. iterate through the sub-directories of “LGG” and read the PAC and CCC values. SUMO utilizes a re-sampling approach, there are multiple values for each clustering rank “k”, crated by calculating metrics on subsets of samples in consensus matrix:
      >np <- import("numpy")
      >sumo_run_dir <- "LGG"
      >dir_names <- dir(sumo_run_dir)[grepl("ˆk[0-9]",
           dir(sumo_run_dir))]
      >pac_tbl <- sapply(dir_names, function(x)
         np$load(file.path(sumo_run_dir, x,
         "sumo_results.npz"),
         allow_pickle = TRUE)$f['pac']) %>%
       as_tibble() %>%
       mutate(rep = row_number()) %>%
       gather("dir_name", "PAC", -rep)
      >ccc_tbl <- sapply(dir_names, function(x)
       np$load(file.path(sumo_run_dir, x,
       "sumo_results.npz"),
       allow_pickle = TRUE)$f['cophenet']) %>%
       as_tibble() %>%
       mutate(rep = row_number()) %>%
       gather("dir_name", "CCC", -rep)
    iii. calculate median, minimum, and maximum for the two metrics corresponding to each clustering rank:
      >metric_tbl <- ccc_tbl %>%
          left_join(pac_tbl,
          by = c("rep", "dir_name")) %>%
          separate(dir_name, c(NA, 'k'),
            sep="k") %>%
       mutate(k=as.factor(as.numeric(k))) %>%
       gather(metric, value, -k, -rep) %>%
       group_by(metric, k) %>%
       summarise(median_value=median(value),
          min_value=min(value),
          max_value=max(value))
    iv. plot the metrics together:
      >metric_tbl %>%
       ggplot() +
        geom_line(aes(x=k, y=median_value,
        color=metric, group=metric), size=1) +
        geom_ribbon(aes(x=k, ymin=min_value,
        ymax=max_value, group=metric, fill=metric),
        alpha=0.3) +
        theme_bw() +
        labs(y="metric value") +
        theme(legend.position = "bottom")

### Step 5: Identifying potential biomarkers

**Timing: 13 h 30 min**

In this step, we identify the features that are predictive of the subtypes determined in the last step. These features are potential biomarkers that can be used to assign new samples to the determined subtypes. We train a tree-based LightGBM model (Ke et al., 2017) using the feature matrices and the assigned labels from the last step, though this sort of feature importance can be determined using other approaches such as support vector machines. We then calculate the Shapley values (Lundberg and Lee, 2017) for each feature in the resulting model. The Shapley value is the average marginal contribution of a feature, and the features with Shapley values greater than one are considered to be important in driving the separation of that cluster.

10. Use following command to run SUMO in the `interpret` mode:
  sumo interpret LGG/k5/sumo_results.npz
  TCGA.LGG.mRNAexp.tsv,TCGA.LGG.met.tsv,TCGA.LGG.miRNAexp.tsv
  LGG_interpret_k5
  a. The required positional arguments of SUMO are:
    i. “interpret” - selected sumo `mode` which determines the importance of features,
    ii. “sumo_results.npz” - path to selected factorization results from the `run` mode of SUMO (here we selected 5 as the optimal number of clusters although 2 is also a good choice, see expected outcomes for more details),
    iii. “infile1,infile2,…” - a comma-delimited list of files containing standardized feature matrices,
    iv. “output_prefix”- prefix of the output files.
  b. Some of the optional arguments in this mode include:
    i. “-log” and “-logfile” - arguments governing the logging level and optional path to save the logging file,
    ii. “-max_iter“ and “-n_folds” - the model parameters that govern the search through hyperparameter space and model cross-validation,
    iii. “-t” number of computational threads.
  c. The outputs of this step are two files with feature importance in form of SHAP (Shapley Additive exPlanations) values calculated using the trained model. The .tsv file contains a matrix of values, with features represented in rows, and the clusters represented in columns. The value of each cell corresponds to the importance of a given feature in that cluster. The .hits.tsv file is a summary of the most important features for each cluster.

### Step 6: Examples of downstream analyses

**Timing: 14 s**

In the previous steps, we identified five different subtypes using the TCGA-LGG data. We also identified genes, methylation probes, and microRNAs that could be potential biomarkers for each of the five subtypes. Now, several downstream analyses can be performed to evaluate the identified subtypes and important features. One such analysis is to determine whether there is a significant difference in prognosis for at least one of the detected subtypes. See expected outcomes for the discussion of results.

11. Load required R packages:

>library(tidyverse)

>library(survival)

>library(survminer)

**Figure 2:**
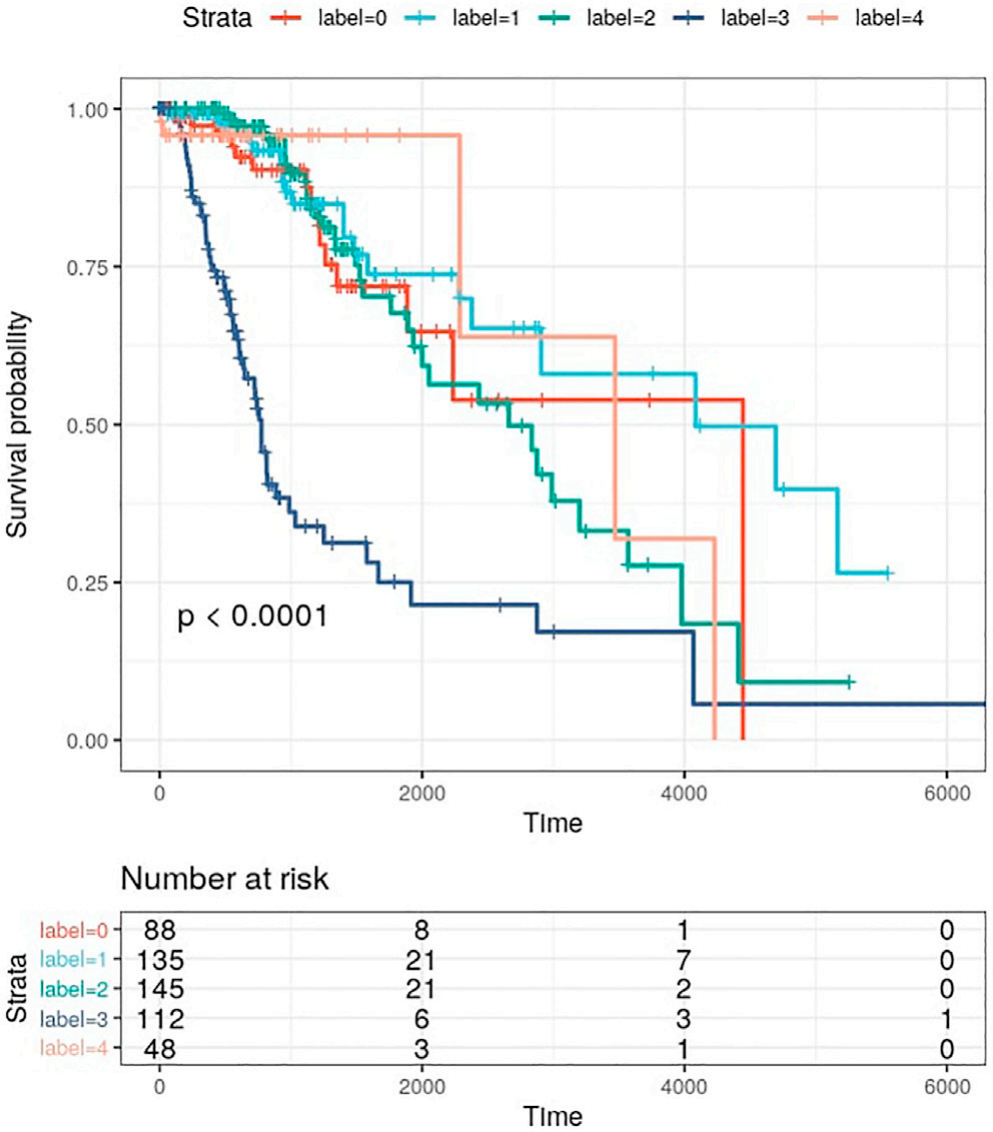
Kaplan-Meier survival analysis for the five subtypes detected by SUMO We report the p-value of the log-rank test.

**Figure 3:**
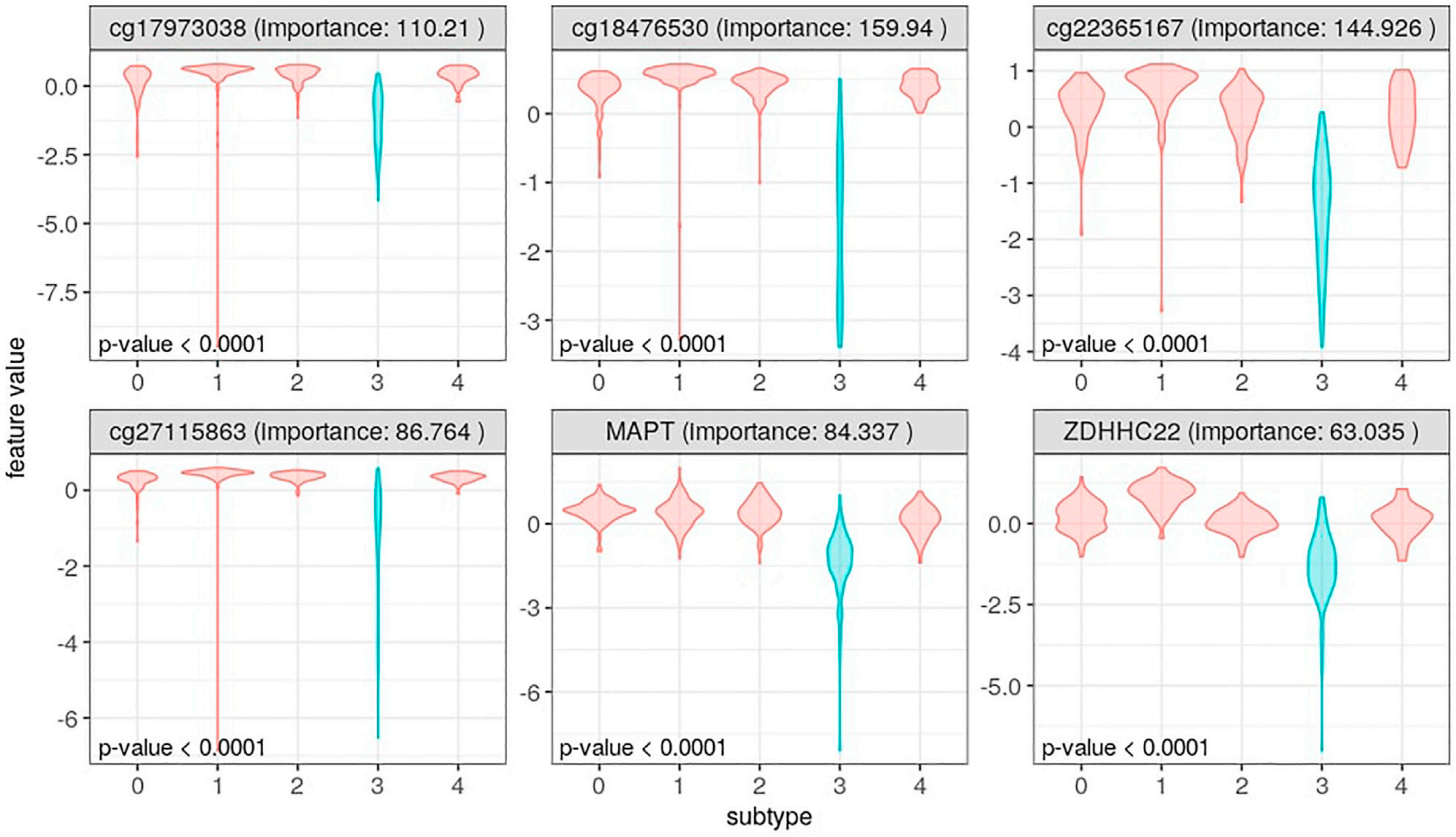
Evaluation of important features for differentiation of subtype 3 We show the top six candidate features identified, by `sumo interpret`. The reported p-value is calculated using the Kruskal-Wallis test.

12. Survival analysis:
  a. Load the sample labels (for the selected number of clusters) produced by SUMO:
    >labels <- read_tsv("LGG/k5/clusters.tsv")
  b. Download the survival data to the working directory (see key resources table) and load it with the following command:
    >data <- read_tsv("survival%2FLGG_survival.txt") %>%
     select(sample, OS, OS.time) %>% right_join(labels, by =
     "sample")
  c. Plot the Kaplan-Meier curves (Figure 2):
    >ggsurvplot(survfit(Surv(OS.time, OS) ∼ label,
     data=data), data=data, palette = "npg", pval = TRUE,
     ggtheme = theme_bw(), risk.table = TRUE)
    ***Note:*** Crossing hazard rates can lead to loss of power in log-rank tests. The assumptions of proportional hazard should be tested using the Schoenfeld residuals and alternative approaches such as weighted log-rank tests should be used in case of violations (Bouliotis and Billingham, 2011).

13. Evaluation of potential biomarkers for a selected subtype of interest:
  a. Load the sample labels, produced by `sumo run`, and corresponding `sumo interpret` results:
    >data <- read_tsv("LGG_interpret_k5.tsv") %>%
     gather(group, importance, -feature)
    >labels <- read_tsv("LGG/k5/clusters.tsv")
  b. Assign the label of a subtype of interest. Here, we supply the label for the cluster with a worse prognosis compared to the other subtypes. In our run, this label was 3, though it might be a different label in another run:
    >selected_label <-3
  c. Find the most important features for the subtype:
    >top_features <- data %>%
     filter(group == paste0("GROUP_",selected_label)) %>%
     arrange(desc(importance)) %>% top_n(6, importance)
  d. Extract the values of top features from pre-processed feature matrices:
    >patterns <- paste('-w', paste0('-e ',
     top_features$feature %>%
      unique(), collapse = ' '), collapse = '')
    >infiles <- c("TCGA.LGG.mRNAexp.tsv",
       "TCGA.LGG.miRNAexp.tsv",
       "TCGA.LGG.met.tsv")
    >data <- NULL
    >for (fname in infiles){
     samples <- colnames(read_tsv(pipe(paste("head -n 1",
        fname)),
       col_types =c(col_character())))
     the_pipe <- pipe(paste("grep", patterns, fname))
     rlines <- readLines(the_pipe)
     if (length(rlines) > 0){
     for(read_line in rlines){
     split_line <- strsplit(read_line, '\t')[[1]]
     feature <- strsplit(split_line[1], '"')[[1]][-1]
     feature_tbl <- tibble(sample=samples,
      feature=feature, fname=fname,
     value=as.numeric(split_line[2:length(split_line)]))
     if(is.null(data)){
      data <- feature_tbl
     } else {
      data <- data %>%
       full_join(feature_tbl,
       by=c("sample","feature","fname","value"))
     }
     }
     }
     close(the_pipe)
     }
    >data <- data %>%
      left_join(labels, by = "sample") %>%
      left_join(top_features, by = "feature")
  e. Confirm if each feature of interest has significantly different values for the cluster of interest using the Kruskal-Wallis test:
    >test_feature <- function(value, label, group){
     the_group <- group[1]
     res <- tibble(value=value,
        label=as.character(label))%>%
      mutate(group=ifelse(label==strsplit(the_group,
       "_")[[1]][-1], the_group, "OTHER_SAMPLES"))
     pval <- kruskal.test(value ∼ group,data=res)$p.value
     return(pval)
     }
    >data <- data %>%
     group_by(feature) %>%
     summarise(pval=test_feature(value, label, group)) %>%
     right_join(data, by = "feature")
  f. Plot values of each feature of interest across the clusters/subtypes (Figure 3):
    >data %>%
     separate(group, c(NA, "group"), sep="_") %>%
     mutate(group=as.numeric(group),
       selected_group=as.factor(label==group),
       label=as.factor(label)) %>%
      ggplot() +
       geom_violin(aes(x=label, y=value,
          fill=selected_group,
          color=selected_group),
          alpha=0.3) +
       geom_text(data=data %>%
          select(feature, group,
           importance, pval) %>%
       distinct(),
       aes(x = -Inf, y = -Inf, label=paste("p-
       value",
       ifelse(pval > 0.0001, round(pval, 4), "<
       0.0001"))), hjust = -0.05, vjust = -0.5, size=4) +
       facet_wrap(paste(feature, "(importance:",
       round(importance, 3),")")∼.,
       scales="free") +
       theme_bw(base_size = 12) +
       theme(legend.position =
       "null") +
       labs(y="feature value", x="subtype")

## Expected outcomes

This protocol presents instructions to integrate the gene expression, miRNA expression, and methylation data from the TCGA-LGG cohort to determine molecular subtypes. The subtyping is based on joint non-negative factorization of patient-similarity networks determined from each data type.

The NMF technique assumes that a single basis vector can represent each cluster and different clusters correspond to different basis vectors. This assumption makes the selection of an optimal factorization rank (the number of discrete subtypes) a challenging problem since each clustering, in theory, presents a viable solution. In practice, we run SUMO for a range of different possible ranks. Along with the cluster labels assigned for each of those ranks, SUMO calculates two internal clustering quality metrics: the proportion of ambiguously clustered pairs (PAC) and cophenetic correlation coefficient (CCC). The optimal rank is characterized by a low PAC and a high CCC value. Supporting the results in Sienkiewicz et al. (2020), the optimal number of subtypes in this dataset is 5, followed by separation into 2 clusters with a higher level of ambiguity (see Figure 1B).

Once the subtypes are identified, the downstream analyses typically focus on the differences between the clusters. The Kaplan-Meier survival analysis for the five clusters identified by SUMO (Figure 2) reveals that one of the subtypes has a significantly worse prognosis compared to the other subtypes (p-value < 0.0001). We then determine the features that separate this subtype from the other subtypes and plot the distribution of the standardized values for the top six features across the various groups. All the features of interest, including four methylation probes (cg17973038, cg18476530, cg22365167, cg27115863) and two genes (MAPT and ZDHHC22), are significantly enriched for the subtype of interest in comparison to the rest of the samples, as determined using the Kruskal-Wallis test (see Figure 3).

## Limitations

SUMO calculates the similarity matrix as a modified Gaussian radial basis function of the Euclidean distance between the samples. This distance is appropriate for several genomic datasets, but it can be limiting if the user wants to integrate sparse or categorical data types. It is possible to decrease the sparsity of the data by performing additional pre-processing steps, such as feature selection (i.e., reduction of feature set, by limiting it to, for example, only known disease-causing genes) or feature transformation (e.g., mapping somatic mutations to known pathways instead of individual genes). Alternatively, if the conversion of non-continuous data types into a continuous data matrix (without the loss of biological information) is possible, such pre-processed data can be integrated using SUMO. For example, somatic mutations can be converted into a continuous dataset using a network propagation approach. The SUMO package documentation (link available in key resources table) includes an example code vignette that details how random walks on a protein-protein interaction network can be used to transform somatic mutation data into a continuous data matrix appropriate for SUMO.

## Troubleshooting

### Problem 1

Errors when installing SUMO from the Python Packaging Index (PyPI). (**Step 1: Package installation**)

### Potential solution

**Figure 4:**
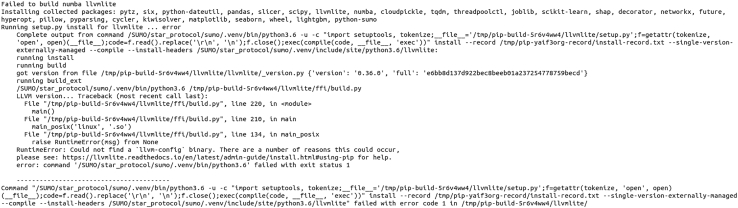
An error message for SUMO installation SUMO encounters errors during the installation of its dependencies: “numba” and “llvmlite”. This is a known issue that appears when the pip (package installer) version is not updated before an attempted SUMO installation.

This error (see Figure 4) may appear during the SUMO installation process if the pip (package installer) version used is outdated. This is a known issue connected to the installation of the “llvmlite” package, which is required by one of SUMO dependencies (“numba”). This problem can be easily solved by upgrading the pip to a 19.0+ version, as described in Step 1. This is the most common installation issue we noted, as the default pip version on most current systems is not sufficient.

### Problem 2

A common error when running `sumo prepare`: data matrix not fully standardized. (**Step 3: Creating similarity networks**)

### Potential solution

**Figure 5:**
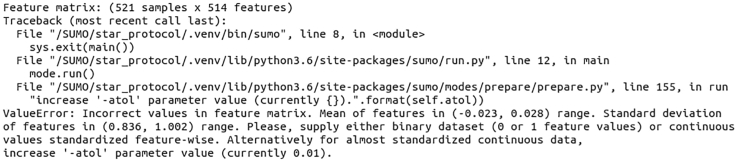
An error message for `sumo prepare` This error informs the user that the input data to SUMO is not standardized. By default, SUMO allows means of features to be in (−0.01, 0.01) and standard deviations in the (0.09, 1.1) range. The tolerance can be increased using the “-atol” parameter.

The recommended data pre-processing for `sumo prepare` includes data standardization. As described in Step 2 of this protocol, we standardize features to have a zero mean and unit variance. After loading the data for the construction of the similarity network, SUMO checks if means and standard deviations of features are within a tolerance level (by default 0.01) of these values. If that is not true, then an error message is returned (see Figure 5) and the execution of the program stops. The error message reports the range of mean and variance for features in the dataset.

The user should reevaluate the standardization to ensure that it has been performed correctly. Sometimes, when the dataset is sparse, e.g., due to a high frequency of missing values, the standardized features can have values for mean and variance across samples that deviate from the expectation of zero and one respectively. In this protocol, the inclusion of miRNA expression, with only 514 features and 3560 missing values, requires that we increase the tolerance range (using the parameter “-atol”) to 0.3, which solves this issue.

### Problem 3

A common error when running `sumo interpret`: the tree model cannot be trained. (**Step 5: Identifying potential biomarkers**)

### Potential solution

**Figure 6:**
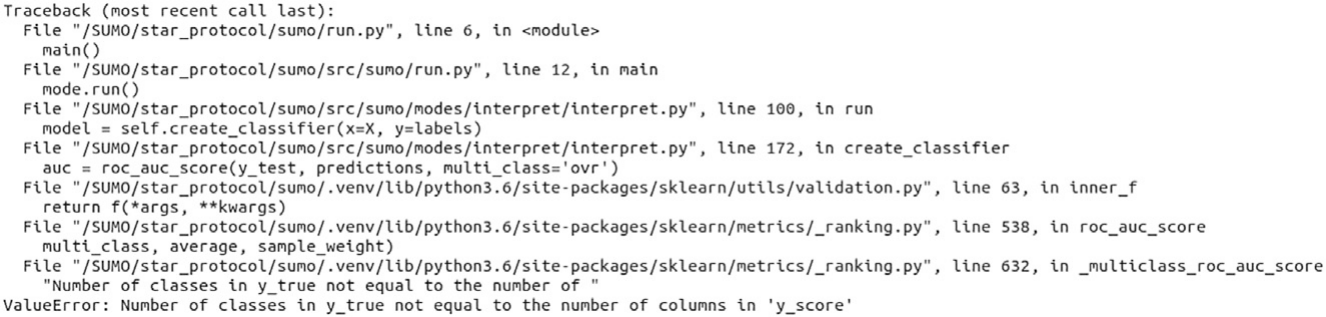
An error message for `sumo interpret` One of the subtypes in the sumo_results.npz file has only one sample. SUMO encounters a problem when attempting to split data into training and testing datasets.

This error (see Figure 6) appears if SUMO detects a cluster with few samples (typically just one or two samples). This causes an issue when splitting the data into training and testing subsets. In such a case with “k” clusters, the user is advised to select “k-1” as the optimal number of clusters.

### Problem 4

Batch effect can influence the determination of subtypes. (**Step 4: Detecting molecular subtypes**)

### Potential solution

Batch effect refers to technical variation or non-biological differences between measurements of different groups of samples. For example, a recent study focused on miRNAs found that plate during library preparation, the sequencing platform (Illumina Hi-Seq vs Illumina Genome Analyzer II), sample purity, and sequencing depth were all drivers of miRNA variability in the Lung squamous cell carcinoma dataset (TCGA-LUSC) (Ibing et al., 2021). These confounders can drive the separation of samples and hence the creation of subtypes if they are not accounted for. We recommend the use of ComBat (Johnson et al., 2007) in data pre-processing to adjust for batch effects when the effects come from known sources.

If you suspect that the identified clusters reflect a known technical artifact, then `sumo evaluate` can be used to compare the labels assigned by SUMO to the known batch labels.

### Problem 5

Errors when loading .npz files in R (with reticulate). (**Step 6: Examples of downstream analyses**)

### Potential solution

Output .npz files produced by SUMO ′prepare` and `run` modes are zipped archives created using the python NumPy module and can be loaded and viewed in python. To read such files in R, as described in this protocol the “reticulate” package is required, which provides an interface to python directly from R. In case, the user has multiple available python versions, some additional configuration of the package may be required. Although the “reticulate” package supports both Python versions, the .npz files created using Python 3 may not load correctly if reticulate is set to use Python 2 version of the NumPy package. We recommend specifying the path to Python (ideally the same version that is used for the rest of the SUMO protocol), before loading the reticulate package. This can be accomplished using the following command:

>reticulate::use_python(python_path, required = TRUE)

We also note that “reticulate” may encounter an error in the “initialize_python” function which prevents Python bindings from being loaded. If this is the case, the designated Python version used by “reticulate” does not have support for shared libraries. If no suitable Python 3 version is available, we recommend re-installing Python 3 from the source with "--enable-shared" option during the package compilation.

## Resource availability

### Lead contact

Further information and requests for resources should be directed to and will be fulfilled by the lead contact, Aakrosh Ratan (ratan@virginia.edu).

### Materials availability

This study did not generate new unique reagents.

## Data Availability

This paper analyzes existing, publicly available data. The download links for the datasets are listed in the key resources table. All code not available directly here can be found in the SUMO documentation (see key resources table). Any additional information required to reanalyze the data reported in this paper is available from the lead contact upon request.
